# IL-37 Isoform A Prevents Collagen-Induced Arthritis in Mice by Modulating the Th17/Treg Balance via IL1R8 Receptors

**DOI:** 10.3390/ijms252312878

**Published:** 2024-11-29

**Authors:** Shuyan Lyu, Zhengyu Fang, Yiping Hu, Miaomiao Zhang, Jiaxin He, Xiaocheng Wang, Juan He, Xu Gao, Hongli Wang, Damo Xu, Qingwen Wang

**Affiliations:** 1 Institute of Immunology and Inflammatory Diseases, Shenzhen Peking University-The Hong Kong University of Science and Technology (PKU-HKUST) Medical Center, Peking University Shenzhen Hospital, Shenzhen 518036, China; shuylv@163.com (S.L.); fangzy796@163.com (Z.F.); huyiping126@126.com (Y.H.); zhangmmwhale@163.com (M.Z.); 2111210603@stu.pku.edu.cn (J.H.); wxcheng@hnu.edu.cn (X.W.); hejuan04@163.com (J.H.); 2211110779@stu.pku.edu.cn (X.G.); 1911210755@pku.edu.cn (H.W.); 2 Institute of Allergy and Immunology, Health Science Center, Shenzhen University, Shenzhen 518060, China

**Keywords:** rheumatoid arthritis, collagen-induced arthritis, IL-37a, IL-1R8, cytokines, Th17 cells, Treg cells

## Abstract

Cytokines play a complex and pivotal role in modulating synovitis in rheumatoid arthritis. Interleukin (IL)-37 is known for its extensive anti-inflammatory properties that set it apart from the majority of other IL-1 family members. However, IL-37a, a member of the IL-37 family, lacks research into rheumatoid arthritis. This research aims to explore the role of IL-37a in regulating T-cell homeostasis in rheumatoid arthritis using the Collagen-Induced Arthritis(CIA) model. IL-37atg mice, a genetically altered strain carrying the human *IL-37a* gene, were used to test the influence of this cytokine on the progression of arthritis. The results show that IL-37atg mice demonstrated a notable reduction in both the incidence and severity of arthritis relative to WT mice. The protective effect was accompanied by lower levels of cytokines in plasma and synovial tissues (such as IL-17A and IL1β) that drive the inflammatory response. The ratio of Th17/Treg decreased in the lymph nodes of IL-37atg mice. However, the knockout of *IL1R8* in IL37atg mice eliminated the effects of IL-37a. Additionally, transcriptomic analysis revealed that Th17 cell differentiation is a key pathway through which IL-37a exerts its protective effects, and experiments confirmed that IL-37a suppresses Th17-polarizing in vitro.

## 1. Introduction

Rheumatoid arthritis (RA), a disabling autoimmune condition, features sustained synovitis, resulting in the progressive deterioration of cartilage and bone, potentially culminating in lasting impairment [1]. The progression of RA is characterized by a complex interplay of cytokines. The existence of various cytokines (e.g., interleukin (IL)-1, IL-6, and transforming growth factor-β (TGFβ)) in the synovial tissue plays a pivotal role in rheumatoid arthritis pathogenesis by promoting the differentiation and proliferation of T cells. Notably, IL-6, IL-1, and tumor necrosis factor (TNF), mainly secreted from macrophages, play significant roles in driving proinflammatory pathways within the synovial tissue. Additionally, RANKL, IL-17, IL-1, and TNF are crucial for osteoclast maturation and activation [2]. These cytokines orchestrate a cascade of immunological events, underscoring their critical roles in both the inflammatory and tissue damage phases of the disease.

Recent investigations have emphasized that Th17/Treg dysregulation promotes the development of RA [3,4]. Studies conducted on animals and peripheral blood samples from RA patients have both confirmed that T helper (Th)17 cells promote the disease progression [5]. Patients with RA exhibit an elevated Th17 cell proportion in their peripheral blood compared with healthy individuals [3]. Furthermore, studies using IL-17-knockout animals have demonstrated that the deficiency of IL-17 reduces the severity of collagen-induced arthritis (CIA) [6]. To maintain immune homeostasis, Treg cells serve an essential function in modulating the activity and function of effector T cells. However, the inhibitory role of Treg cells is hindered in RA [4]. The study found that RA patients exhibited a lower percentage of regulatory T (Treg) cells in their peripheral blood samples compared with healthy individuals [3]. Interestingly, the biological drug tocilizumab helps restore the Th17/Treg balance in RA patients [7].

IL-37 plays a potential anti-inflammatory role in rheumatoid arthritis (RA) [8]. Five distinct splice variants of IL-37 have been discovered, namely IL-37a–e [9]. IL-37b, which contains the most exons (exon 1–2 and exon 4–6), has been extensively studied, revealing its significant impact on inhibiting inflammation. In vitro investigations have revealed that IL-37b can downregulate various proinflammatory cytokines, including TNFα, IL1β, IL1α, IL6, IL8, and IL23 [10]. The extracellular forms of IL-37b require IL-18Rα and IL-1R8 receptors to exert their effects [11,12]. Additionally, the intracellular form of IL-37b undergoes processing by caspase-1, and the mature form of IL-37b relies on the nuclear translocation of SMAD3 to act as a transcription factor [13,14]. IL-37d, another isoform consisting of exon 1 and exon 4–6, has also been shown to act as an immunosuppressive agent in both in vitro studies and in vivo studies. IL-37d was demonstrated to downregulate proinflammatory factors in an IL1R8-independent manner in mouse peritoneal macrophages. However, it can inhibit P65 phosphorylation to suppress the activity of NF-κβ in adipose-derived stem cells, with this inhibition being dependent on IL-1R8. Moreover, IL-37d has been found to inhibit DSS-induced colitis in an IL1R8-dependent manner [15,16,17]. Currently, research into IL-37a, IL-37c, and IL-37e is limited. Given that IL-37c and IL-37e lack exon 4, they are predicted to be non-functional proteins [18,19]. Nonetheless, existing research has suggested the potential of IL-37e as a disease biomarker [20]. Particularly, IL-37a, which has often been overlooked, is now being re-evaluated. Wei et al. have demonstrated that IL-37a not only has extracellular functions dependent on IL-1R8 but also acts as a nuclear factor via IL-1R8-independent mechanisms to inhibit inflammation in a mouse model of lethal lipopolysaccharide (LPS)-induced shock [21,22]. However, research into the functions and mechanisms of IL-37a in RA is still lacking. In this research, we utilized IL-37a transgenic (IL-37atg) mice to evaluate the efficacy of IL-37a in preventing collagen-induced arthritis. Additionally, IL-1R8-deficient IL-37atg (IL37atg, IL1R8-/-) mice were used to investigate whether IL-37a relies on the IL-1R8 receptor pathway. We were objective to unveil the important role of IL-37a in modulating immune homeostasis, providing potential therapeutic approaches for RA.

## 2. Results

### 2.1. IL-37a Is Expressed in Peripheral Blood Mononuclear Cells (Pbmcs) from RA Patients and RA-Fibroblast-like Synoviocytes (FLSs) and Is Markedly Upregulated by Tnfα

**Figure 1:**
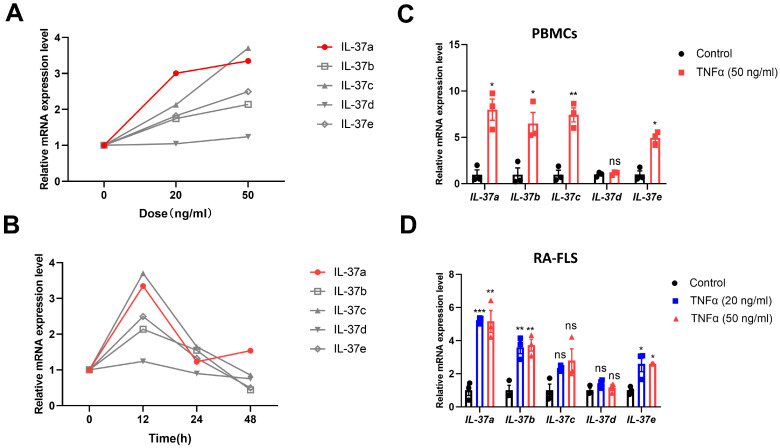
Different expression patterns of IL-37(a–e) in PBMCs from RA patients and RA-FLSs. (**A**,**B**) PBMCs were stimulated with the varying dose (**A**) of TNFα for 12 h and stimulated for the indicated times (**B**) with a fixed dose of TNFα (50 g/mL). RNA extraction was performed to obtain the whole collection of RNA molecules, and quantitative polymerase chain reaction (qPCR) was used to analyze the levels of IL-37(a–e) mRNA. (**C**,**D**) PBMCs (**C**) were stimulated with TNFα for 12 h, and RA-FLSs (**D**) were stimulated with TNFα for 24 h. Control (n = 3), TNFa group (n = 3). The control group served as an untreated comparison group. The data are presented as the means ± SEM. * *p* < 0.05, ** *p* < 0.01 and *** *p* < 0.001 vs. the control group.

There have been limited reports about the different expression patterns of five IL-37 isoforms, particularly within the context of RA. Our initial investigation employed quantitative PCR to study five IL-37 isoform expression patterns in PBMCs together with RA-FLSs. TNFα was used to activate the immune status of PBMCs and RA-FLSs. Earlier research has demonstrated that stimulating cells with 20 ng/mL TNFα for 20 h leads to a significant upregulation in IL-37 expression [10]; we designed an experiment with varying stimulation doses (0, 20, and 50 ng/mL) and time intervals (0, 12, 24, and 48 h). Our results demonstrated that the expression of IL-37(a–d) in TNFα-stimulated PBMCs raised in a dose-dependent manner at every time point, with the highest levels observed at 12 h (Figure 1A,B). Consequently, we selected a dose of 50 ng/mL and a stimulation time of 12 h for further experiments on multiple PBMC samples. The levels of IL-37a, IL-37b, IL-37c, and IL-37e mRNA were significantly elevated in PBMCs after a 12 h treatment with 50 ng/mL TNFα (Figure 1C). Similarly, IL-37a, IL-37b, and IL-37e expression in RA-FLSs exhibited comparable trends following TNFα stimulation (Figure 1D). Notably, among these isoforms, IL-37a showed the most significant change, with an increase of more than 5-fold in both PBMCs and RA-FLSs (Figure 1C,D). Additionally, a report has indicated a significant alteration in the expression of all IL-37 isoforms following the stimulation of PBMCs with lipopolysaccharide (LPS) [21]. We have replicated this experiment by stimulating PBMC derived from RA patients with LPS. In PBMCs, the levels of IL-37(a–e) mRNA were all upregulated following a 6 h stimulation with 1 μg/mL LPS (Figure A2A), with IL-37a showing the most significant change (more than 20-fold). Compared with TNFα stimulation, stimulation with LPS led to a more substantial elevation in the expression of IL-37 isoforms in PBMCs. The data collectively emphasize the pivotal role of IL-37a, a key isoform that is highly responsive to the proinflammatory cytokine TNFα, in the context of RA. Moreover, our results showed that there were no significant differences in the expression levels of IL-37(a, b, d, e) in PBMCs from RA patients, both before and after stimulation, compared with healthy controls (HC) (Figure A2B,C). However, in resting PBMCs, the expression level of IL-37c in the RA group was significantly lower than that in the HC group (Figure A2C).

### 2.2. IL-37 Isoform A Is Effective in Preventing Collagen-Induced Arthritis and IL1R8 Deficiency Can Diminish the Effect of IL-37a

**Figure 2:**
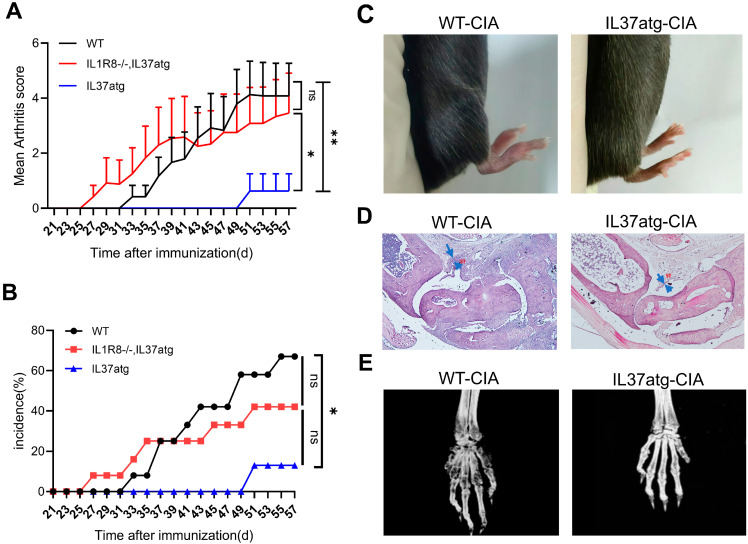
IL-37a prevents the pathology of CIA and is dependent on the receptor IL1R8. (**A**,**B**) The arthritis score (**A**) and incidence (**B**) of CIA in WT mice (n = 12), IL-37atg mice (n = 8), and IL-37atg with IL1R8-/- mice (n = 12). The arthritis scores are presented as the means + SEM. (**C**) Representative image of the swollen joint in the CIA model. (**D**) H&E staining of representative ankle joint sections. The blue arrow indicates the synovial lining layer. ST: synovial tissue. Magnification: 40×. (**E**) Representative micro-CT images showing bone damage in the paws of each group of mice. * *p* < 0.05 and ** *p* < 0.01 vs. the WT group.

The role and regulatory mechanisms of IL-37a in RA remain poorly understood. To investigate this possibility, we utilized transgenic mice expressing human IL-37a. We induced autoimmune arthritis in mice and assessed the histological features of CIA, which closely resembles RA. Notably, the incidence and severity of arthritis were markedly lower in IL-37atg mice than in WT mice (Figure 2A,B). As shown in Figure 2C, the WT mice displayed marked redness and swelling in their paws, while the IL-37atg group showed minimal occurrences. These findings were further supported by HE staining and micro-CT analysis, which revealed that IL-37a expression significantly reduced synovial inflammation in the ankle joint and bone erosion in the front paw (Figure 2D,E). As indicated by the blue arrow in Figure 2D, after the CIA model was established, the synovial lining layer of the ankle joints in wild-type mice showed significant thickening. Additionally, micro-CT scanning was performed on the forelimbs of the mice. The analysis of trabecular parameters revealed that the bone volume-to-tissue volume ratio (BV/TV) (Figure A3A) was significantly reduced in the wild-type (WT) mice. There was also a significant decrease in the number of trabecular bones (Tb. N) (Figure A3C) and a significant increase in the average distance between trabecular bones (Tb. Sp) (Figure A3D). However, the thickness of the trabecular bones (Tb. Th) (Figure A3B) did not show any significant changes. These findings suggest that WT mice experienced significant bone loss compared with the IL-37a knockout group. To study whether IL-1R8 participates in the suppression of arthritis mediated by IL-37a, we generated IL-37a transgenic mice with an IL-1R8 receptor knockout (IL-37atg, IL-1R8-/-). As shown in Figure 2A,B, IL1R8 deficiency diminished the activity of IL-37a in collagen-induced arthritis. Following the IL-1R8 receptor knockout, the mean score of arthritis was notably greater than the IL-37atg mice. Moreover, IL-17a and IL-2 plasma levels were notably elevated in IL-37atg, IL-1R8-/- mice (Figure A4A,B) compared with IL-37atg mice, indicating that IL-1R8 is essential for IL-37a-mediated suppression of arthritis and inflammatory responses.

### 2.3. IL-37atg Mice Have Lower Levels of Proinflammatory Cytokines in the Plasma and Synovial Tissues

**Figure 3:**
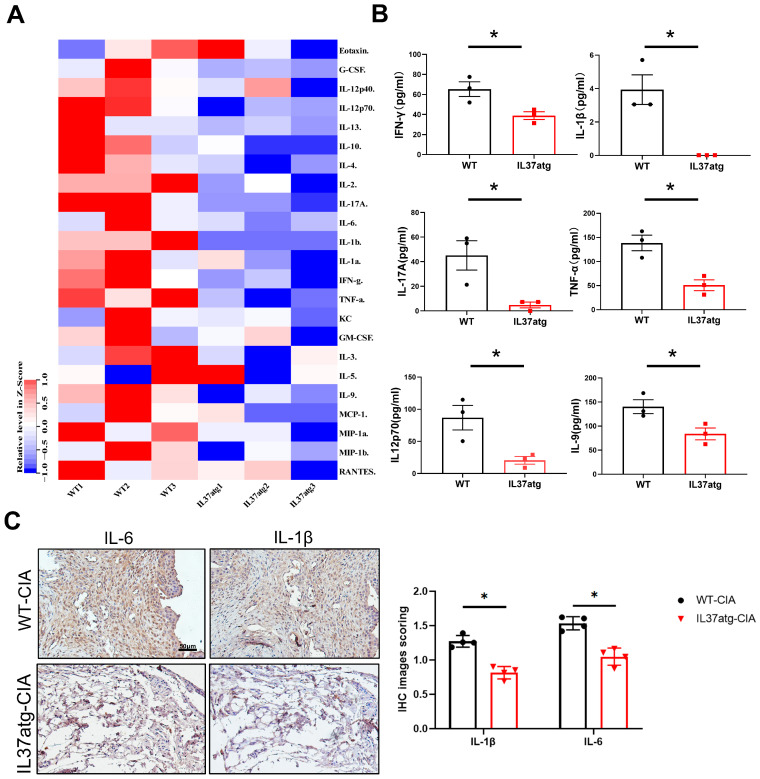
The function of IL-37a is associated with decreased levels of the proinflammatory cytokines. (**A**) Heatmap showing the plasma cytokine and chemokine profiles of WT mice with CIA and IL-37atg mice with CIA. WT (n = 3), IL37atg (n = 3). (**B**) Comparing the concentrations of INF-γ, IL-17A, IL-1β, TNF-α, IL-9, and IL12p70 in the IL-37atg group and WT group. (**C**) Ankle joints underwent immunohistochemical staining for IL-1β and IL-6. WT (n = 4), IL37atg (n = 4). IHC image scoring using the IHC Profiler by ImageJ, version 2.3.0 [25]. * *p* < 0.05 vs. the WT group.

Plasma levels of different cytokines were assessed in mice of two different gene types using an array of 21 cytokines and the Luminex 200 system. Data normalization was performed by rows. Overall, the heatmap indicated that the IL-37atg group had reduced levels of various inflammatory factors compared with the WT group (Figure 3A). Among these, INF-γ, IL-17A, IL-1β, TNFα, IL-9, and IL12p70 showed a notable decrease in the IL-37atg mice (Figure 3B). The levels of anti-inflammatory cytokine IL-10 [23] were slightly lower in IL-37atg mice than in WT mice, suggesting that IL-10 did not contribute to the immunosuppressive effect of IL-37a [21]. During the progression of RA, macrophages and synovial fibroblasts secrete cytokines (IL-1β and IL-6) [24], which are abnormally expressed in synovial tissue, and they synergistically promote the skewing of Th17 cells. Given the distinct pathological features of synovial inflammation observed in CIA mice, we also sought to explore how IL-37a might influence these proinflammatory cytokines in the local synovium of the joints. Using immunohistochemistry, we examined the levels of cytokines IL-1β and IL-6 in mouse joint tissue. Immunohistochemical scoring was performed based on the grayscale values of the images [25]. The IL-37atg mice exhibited significantly lower levels of IL-1β and IL-6 compared with the wild-type (WT) mice, as indicated by a notably lower positive area in the IL-37atg group. Moreover, we observed that the abundance of IL-6 in the joint tissue was higher than that of IL-1β (Figure 3C). These findings indicated that IL-37a exerts its immunosuppressive effects by downregulating multiple proinflammatory cytokines, both systemically and locally, in the synovium. 

### 2.4. Th17 Cell Skewing Occurs in the Lymph Nodes of Wild-Type Mice with Collagen-Induced Arthritis (CIA), While the Balance Is Maintained in IL-37a Transgenic Mice with CIA

**Figure 4:**
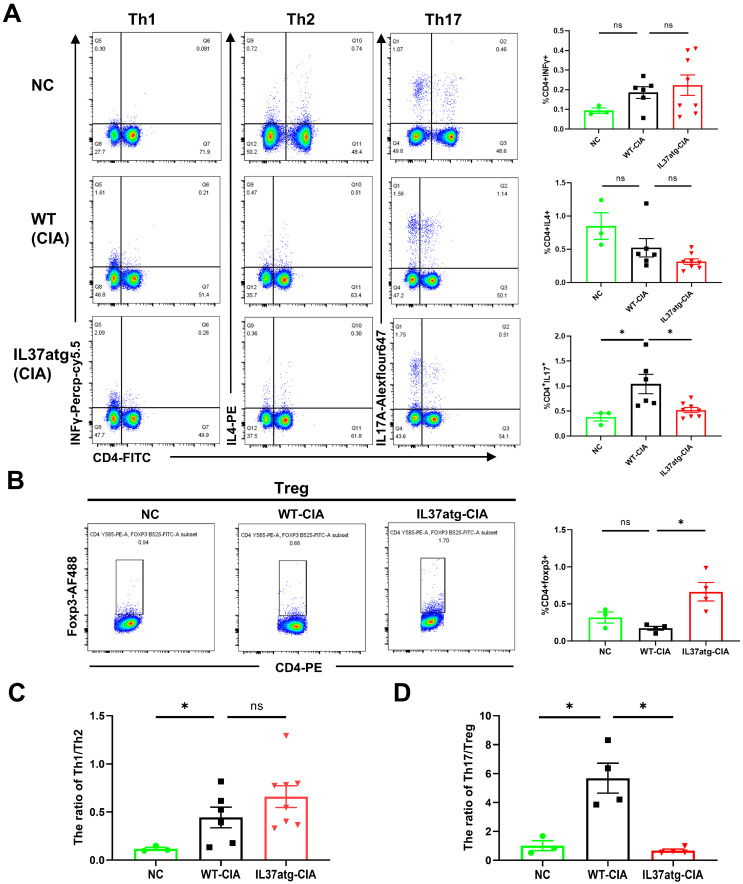
The expression of IL-37a can modulate the Th17/Treg ratio in CIA. (**A**) Determine the proportion of Th1/Th2/Th17 cells among lymph node cells by flow cytometry. And quantification of CD4+INFγ+ cells, CD4+IL4+ cells, and CD4+IL17A+ cells. NC (n = 3), WT-CIA (n = 6), IL37atg-CIA (n = 8). (**B**) Determination and quantification of Treg cells (CD4+FOXP3+) in lymph node cells. The positive cell population is within the black box. NC (n = 3), WT-CIA (n = 3), IL37atg-CIA (n = 4). (**C**) The ratio of Th1/Th2 was determined. (**D**) The ratio of Th17/Treg was determined. NC: the normal control was untreated wild-type mice. * *p* < 0.05 vs. the WT group.

Rheumatoid arthritis is attributed to disruptions in the equilibrium of T-cell subsets [26]. Meanwhile, the CIA model is characterized by a robust and continuous T-cell response [27]. Therefore, through flow cytometry, we assessed the proportions of T-cell subsets in lymph node cells (LNs) to investigate the mechanism involved in the IL-37a-mediated suppression of arthritis. Figure A5A,B shows the flow cytometry strategy. Flow cytometry results showed a reduced proportion of Th17 cells in the IL-37atg mice with CIA relative to the WT mice with CIA (Figure 4A). Conversely, the proportion of Tregs was increased in IL-37atg mice (Figure 4B). Moreover, the WT-CIA mice exhibited an increased Th17/Treg ratio compared with the normal control mice, suggesting an imbalance of Th17/Treg in CIA mice (Figure 4D). The transgene of IL37a decreased the Th17/Treg ratio in CIA mice (Figure 4D). However, the Th1 to Th2 cells ratio did not show notable variances between the WT mice and the IL-37atg mice (Figure 4A,C). In summary, these findings indicated that IL-37a exerts its anti-arthritic effects by adjusting the Th17/Treg balance.

### 2.5. IL-37atg Mice Exhibit Reduced Rorc (RAR-Related Orphan Receptor Gamma) mRNA Expression in Lymph Nodes Compared with WT Mice, and IL-37a Suppresses the Naïve CD4+ T Cells Differentiation into Th17 Cells In Vitro

**Figure 5:**
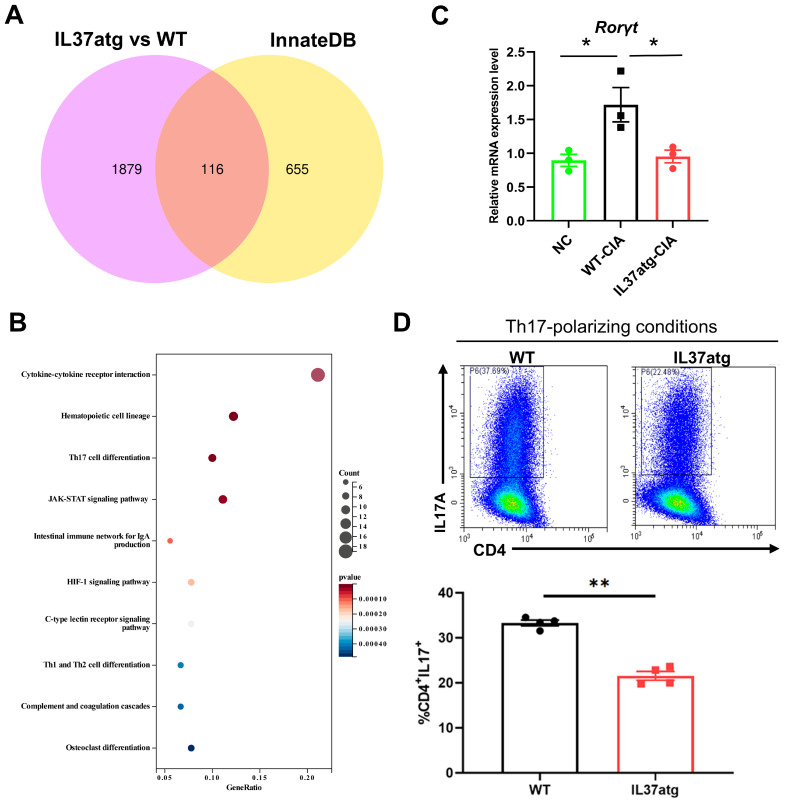
IL-37a inhibits Th17 cell differentiation. (**A**) The InnateDB database was utilized to screen for DEGs associated with the innate immune response. (**B**) KEGG enrichment analysis of the innate immune genes regulated by IL-37a. (**C**) Rorc mRNA expression in single-cell suspensions from draining LNs was measured by qPCR, and the NCs were not used to treat the WT mice (n = 3). (**D**) Flow cytometry and quantification of CD4+IL17+ cells among sorted naïve T cells from WT mice and IL-37atg mice under Th17-polarizing conditions. The positive cell population is within the black box. WT (n = 4), IL37atg (n = 4). * *p* < 0.05 and ** *p* < 0.01 vs. the WT group.

Transcriptomic analysis revealed the potential pathways through which IL-37 regulates arthritis. Using the InnateDB database, we screened DEGs related to the innate immune response. We identified 116 genes regulated by IL-37a (Figure 5A). Additionally, Figure 5B illustrates the ten most significant KEGG signaling pathways associated with immune system function and signal transduction processes, with the cytokine–cytokine receptor interaction pathway being the most affected. Based on previous studies, we focused on Th17 cell differentiation. The expression of RORγt (RAR-related orphan receptor gamma) is essential for preserving the lineage stability and pathogenicity of mature Th17 cells [28,29]. The continuous expression of FOXP3 (Forkhead box P3) is crucial for preserving the lineage identity of mature Treg cells [30]. In this study, we found that the levels of Rorc (Figure 5C) mRNA in mouse LNs were lower in IL-37atg mice with CIA than in WT mice with CIA. Nonetheless, the level of Foxp3 mRNA did not show a significant difference between IL-37atg mice and WT mice (Figure A6A). The ratio of Rorc to Foxp3 mRNA was also significantly lower in the IL-37atg mice compared with the WT mice (Figure A6B). Furthermore, the mRNA level of Il-17a in CIA mice showed a positive correlation with the ratio of Rorc mRNA to Foxp3 mRNA, while this trend was not detected in the untreated group (Figure A6C). Next, we examined whether IL-37a had any influence on Th17-polarizing. The results indicated IL-37a inhibited the Th17 cell differentiation, with the IL-37atg group displaying a notably reduced frequency of Th17 cells compared to the WT group (Figure 5D). In summary, these findings demonstrated that IL-37a regulates inflammatory arthritis through its influence on the Th17 cell lineage.

## 3. Discussion

IL-37a, a member of the IL-37, is lacking research into its function and mechanism in rheumatoid arthritis. Our study revealed that IL-37a is a functional cytokine that impairs CIA, with the effects of IL-37a attributed to a reduction in various inflammatory cytokines and the maintenance of the Th17/Treg balance in the lymph nodes surrounding the joints. Moreover, our research offers initial proof that IL-37a relies on IL1R8 at least partly to exert its suppressive effects on arthritis.

Previous research has shown that IL-37a is present in the tissues of the human testis, colon, placenta, lung, lymph node, and brain [9]. Our results showed that IL-37a exhibited minimal expression in PBMCs but was significantly upregulated (up to 20-fold) following lipopolysaccharide (LPS) stimulation and more than 5-fold after TNFα stimulation. The substantial increase in IL-37a production in response to these factors highlights its crucial role in innate immune reactions. In RA, TNF-α activates synovial fibroblasts, which can prompt cartilage and bone destruction [31]. Raising the TNFα concentration from 20 ng/mL to 50 ng/mL did not lead to an increase in IL-37 expression in RA-fibroblast-like synoviocytes (RA-FLSs). These findings suggested the IL-37 mRNA expression in RA-FLSs is restricted. Additionally, our study revealed that IL-37d does not respond to TNFα stimulation in either RA-FLSs or PBMCs. However, it does respond to LPS stimulation, leading to a rise in mRNA levels. The reasons behind this difference remain to be determined in future studies. In addition, our results showed that PBMCs from RA patients and healthy individuals have similar responses to LPS and TNFα. This suggests that the primary reason for disease progression is the increase in proinflammatory cytokines, such as IL-6, TNF-α, and IL-17A, rather than a decrease in the production of the anti-inflammatory cytokine IL-37.

The CIA model is a standard model for preclinical research into rheumatoid arthritis (RA) and effectively reflects the pathological changes and development process of RA [32]. Therefore, the CIA model was utilized to assess the impact of IL-37a on RA. We induced autoimmune arthritis in both WT mice and in IL-37atg mice. Our results revealed that various plasma cytokines levels were decreased in IL-37atg mice relative to WT mice. It is well known that inflammatory cytokines can directly promote the immune processes associated with the progression of RA [2]. TNFα, primarily derived from macrophages and monocytes, exerts multiple effects on RA. It enhances the phagocytic capacity of macrophages, promotes neutrophil migration across the endothelial cell layer, stimulates synovial fibroblast proliferation, and activates osteoclasts [33]. IL-1β is widely recognized as a crucial proinflammatory cytokine responsible for inducing bone and cartilage damage in rheumatoid arthritis (RA) [34]. IFN-γ enhances chemokine production and activates macrophages and fibroblast-like synoviocytes (FLSs), leading to increased antigen presentation [33]. IL-17 promotes the development of joint inflammation in both human and animal models [5]. Additionally, IL-9 promotes the function of both Th17 and Treg, according to studies [35,36]. IL12p70 was found to promote Th1-polarizing from naïve CD4+T cells [37]. Our findings indicated that IL-37a exerts its suppressive effect on CIA, which might be attributed to inhibiting multiple inflammatory cytokines.

The Th17/Treg cell balance is disrupted in RA patients [3]. Interestingly, our study suggested that IL-37a helps to maintain the balance, with a decreased ratio of Th17/Treg cells in IL-37atg mice relative to WT mice in the CIA model. As shown in previous studies, TGF-β can stimulate the production of Foxp3, promoting Treg differentiation, while inhibiting Th17 cell differentiation by antagonizing the function of RORγt. However, the presence of IL-6 can counteract this process and shift the T-cell differentiation toward the Th17 lineage (CD4+IL17+) [38,39]. IL-1β can also promote the population of Th17 cells together with IL-6 [39,40]. Immunohistochemistry revealed that IL-6 and IL-1β levels in the joint tissue were markedly lower in IL37atg mice compared with WT mice. Additionally, the circulating levels of IL-17a and IL-1β in the blood were markedly lower in IL-37atg mice. These findings might be involved in the decreased ratio of Th17/Treg cells in IL-37atg mice compared with WT mice. Despite these insights, the specific sites in vivo that promote the shift to Th17 cell differentiation are unclear. Furthermore, we were curious about the pathways through which IL37atg mice exhibit a lower Th17/Treg ratio than WT mice. Our transcriptomic analysis revealed that the DEGs between IL37atg mice and WT mice were significantly enriched in the Th17 cell differentiation pathway. Moreover, IL-37atg mice exhibit reduced Rorγt expression in lymph nodes relative to WT mice. Importantly, Th17 cells depend on Rorγt expression to sustain their lineage stability and pathogenic potential [28]. Strikingly, subsequent investigation revealed that naïve T cells from IL-37a transgenic mice showed a weakened capability to differentiate into Th17 cells, indicating that IL37a can directly inhibit the Th17 cell differentiation. A previous study revealed the inhibitory effect of IL-37 on Th17 cell proliferation. In the abovementioned study, recombinant IL-37 was added into culture conditions [41], implying that IL-37 exerts its direct effects on the proliferation of Th17 cells through a receptor-dependent pathway. These findings highlight the complex function of IL-37 in modulating Th17 cell differentiation or proliferation to reduce the proportion of Th17 cells, especially considering IL-37 consists of five isoforms in humans.

A previous study reported that the exon 4–exon 6 sequence in IL-37a can bind to the IL1R8 receptor [12]. In our study, we confirmed that the effects of IL-37a were attenuated after IL1R8 receptor knockout, indicating that IL-37a at least partly relies on the IL1R8 receptor to inhibit collagen-induced arthritis in mice. In addition to its receptor-dependent functions, previous research has revealed that IL-37a is a nuclear cytokine and upregulates the transcription of Pparg in vitro, leading to the inhibition of proinflammatory cytokine production triggered by LPS stimulation [3]. Due to the complex nature and multi-stage characteristics of RA, various cell types contribute to the progression of this disease. IL37a may inhibit the development of arthritis through two mechanisms: one pathway is dependent on the IL1R8 receptor, while the other involves direct regulation by entering the cell nucleus, a process that does not rely on membrane-bound receptors. The importance of these actions may differ across various cell types. For example, our results showed that the expression level of the Foxp3 in lymph node tissues was significantly lower in IL37atg, IL1R8-/- mice compared with the IL-37atg mice, while the level of Rorγt showed no significant change (Figure A4C,D). This suggests that IL-37a has a different degree of dependence on the IL1R8 receptor in Th17 cells compared with Treg cells. Our transcriptome sequencing results also support this hypothesis, revealing 972 and 1031 genes that exhibited differential expression in response to IL-37a regulation in a receptor-dependent and receptor-independent manner, respectively (Figure A4E,F). These findings highlight the specificity of IL-37a, a cytokine with dual functional pathways, suggesting its multiple regulatory roles in complex autoimmune diseases.

Based on previous studies focusing on IL-37b [10,11,12,13,41,42], we sought to compare the differential effects of IL-37a and IL-37b. Research has demonstrated that IL-37a exerts a more significant protective effect than IL-37b in a lethal LPS shock model, with IL-37a transgenic mice showing an increase in the survival rate of more than 20% [21]. However, in the CIA model, a study has explored the effect of IL-37 on CIA by injecting mouse joint cavities with an exogenous adenovirus-packaged recombinant human IL-37 gene plasmid [41]. Due to the variations in experimental designs, it is currently not feasible to directly compare the distinct protective effects of IL-37a and IL-37b on collagen-induced arthritis.

## 4. Materials and Methods

### 4.1. Cell Culture

Fresh human peripheral blood samples were used to obtain peripheral blood mononuclear cells (PBMCs) by utilizing Ficoll-Paque PLUS solution (17-1440-03, GE Healthcare, Chicago, IL, USA). Next, PBMCs were cultured using RPMI 1640 complete medium. RA-FLS, which were generated in our previous study, were successfully derived from synovial tissues of rheumatoid arthritis (RA) patients [43,44]. Dulbecco’s Modified Eagle Medium (DMEM), including a 10% concentration of fetal bovine serum, was used to culture primary RA-FLS. Cell cultures were maintained in an incubator that provided a stable temperature and atmosphere (37 °C, 5% CO_2_). PBMCs and RA-FLSs were exposed to different doses of human TNF-α (HZ-1014-GMP, Proteintech, Rosemont, IL, USA).

### 4.2. Obtaining IL-37atg Mice and IL-37atg, IL1R8-/- Mice

**Figure A1:**
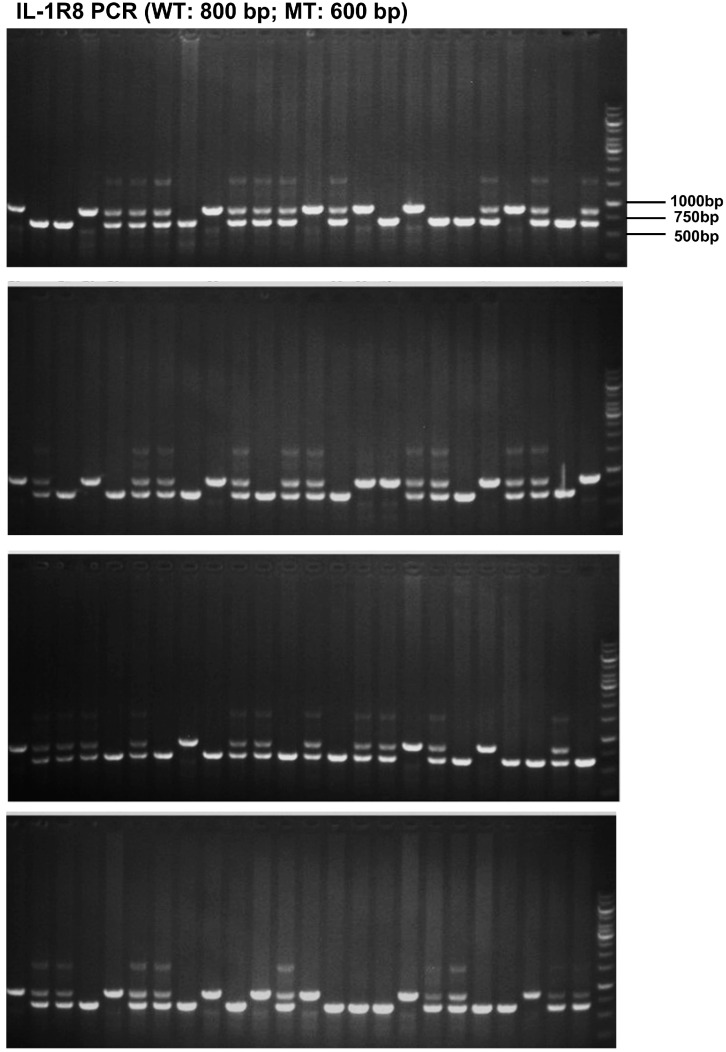

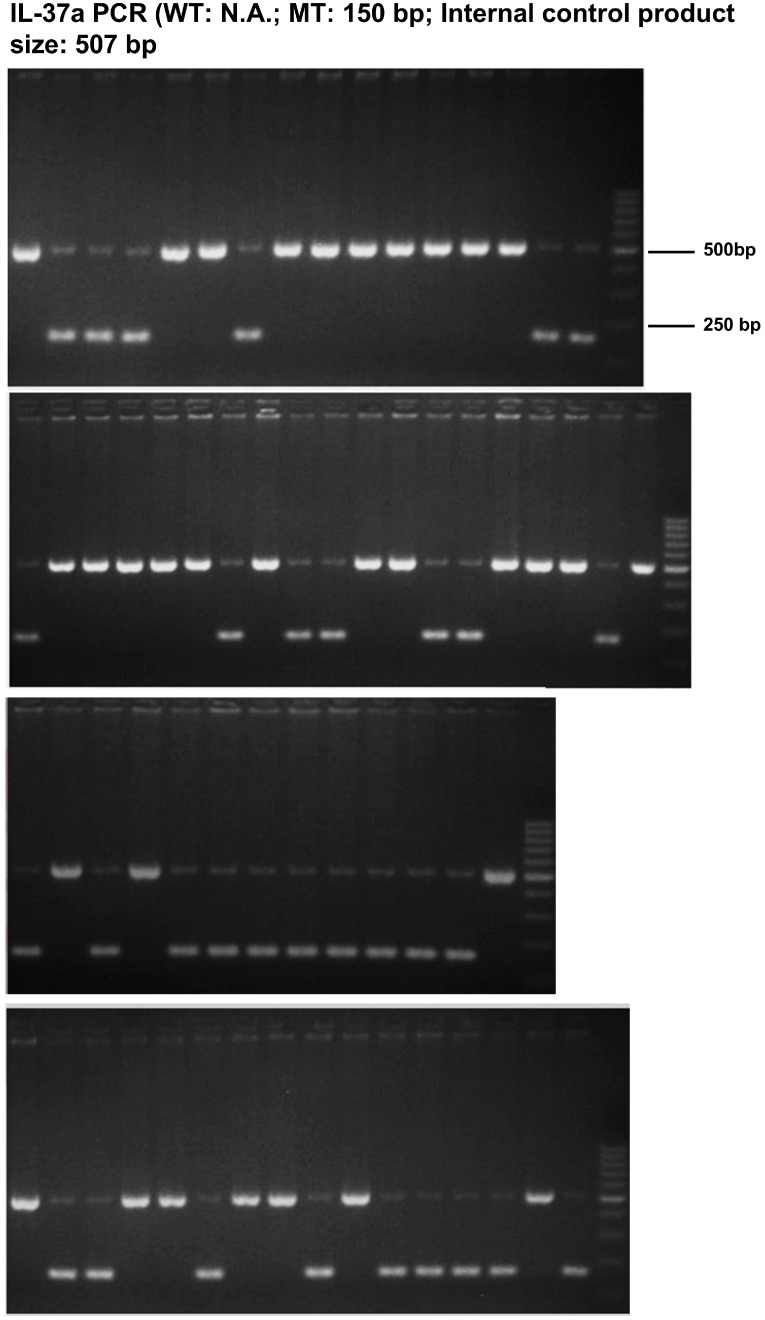
The genotyping of mice by PCR. Primers for IL-1R8 PCR: (Forward: 5′-GAAGGATGAGGGAGGCAGG-3′, Reverse: 5′-GGAGAGATTGCAGATTTAGGCTAAGTG-3′), for IL-37a PCR: (Forward: 5′-ATGTCAGGCTGTGATAGGAGGGAAACAG-3′, Reverse: 5′-TGCTATGAGATTCCCAGAGTCCAGGACC-3′, Internal control PCR primer F: 5′-CTATCAGGGATACTCCTCTTTGCC-3′, Internal control PCR primer R: 5′-GATACAGGAATGACAAGCTCATGGT-3′). For IL-1R8: wild-type mice show one band with 800 bp, heterozygotes show two bands with 600 bp and 800 bp, and homozygotes show one band with 600 bp; for IL37a: wild-type mice only have one band with 507 bp, which is an internal control product, and IL37atg mice show a target band with 150 bp.

**Figure A2:**
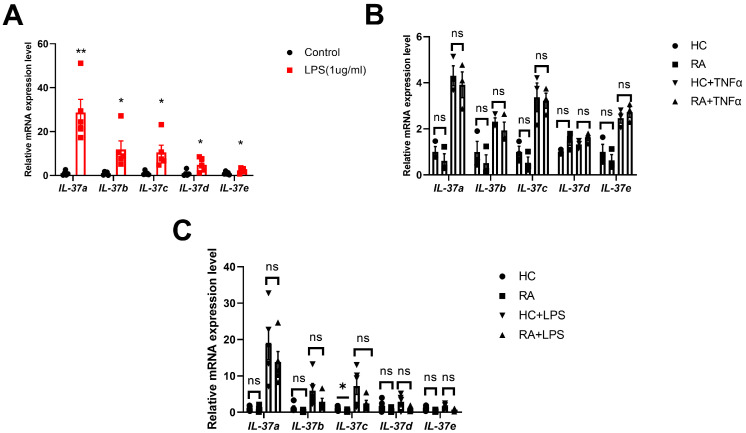
The expression patterns of IL37(a–e) in healthy people compared with RA patients. (**A**) IL37(a–e) was significantly induced by LPS in PBMCs from RA patients. n = 5. (**B**) PBMCs from healthy control (HC) and RA patients were stimulated with TNFα (50 ng/mL) for 12 h. HC (n = 3), RA (n = 3). (**C**) PBMCs from healthy control (HC) and RA patients were stimulated with LPS (1 μg/mL) for 6 h. HC (n = 5), RA (n = 5). The expression of five IL37 isoforms was measured by qPCR. The control group was untread. The data represent the mean ± SEM and are derived from at least three different experiments. * *p* < 0.05 and ** *p* < 0.01 vs. the control group.

**Figure A3:**
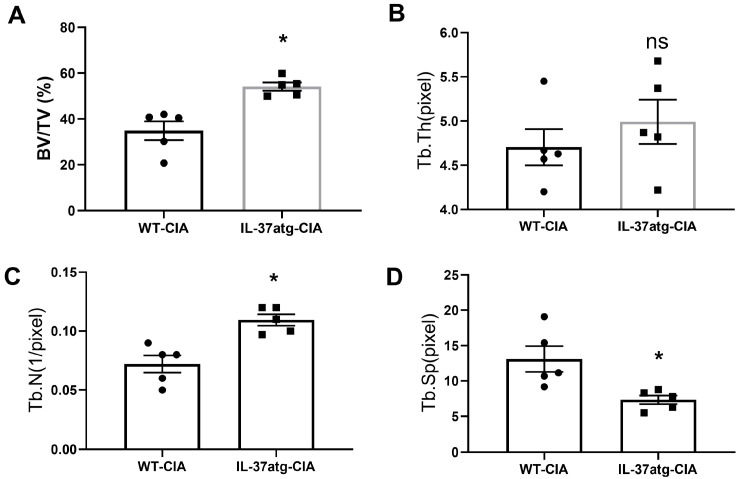
Analysis of parameters related to bone imaging from micro-CT scans. (**A**) The bone volume-to-tissue volume ratio (BV/TV). (**B**) The thickness of the trabecular bones (Tb.Th). (**C**) The number of trabecular bones (Tb. N). (**D**) The average distance between trabecular bones (Tb. Sp). * *p* < 0.05 vs. the WT group.

**Figure A4:**
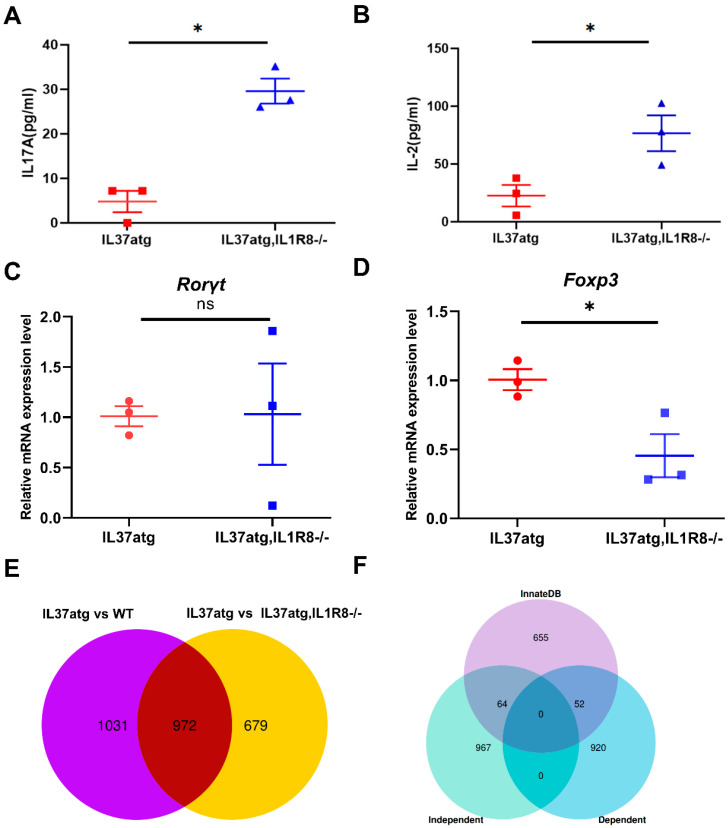
IL1R8 deficiency can diminish the effect of IL-37a. (**A**,**B**) The concentration of inflammatory cytokines IL-17A (**A**) and IL-2 (**B**) in plasma of the IL-37atg group compared with the IL-37atg × IL-1R8-deficient group. (**C**,**D**) Rorγt (**C**) and Foxp3 (**D**) mRNA expression in single-cell suspensions from draining LNs was measured by qPCR (**E**) Euler diagram separated differentially expressed genes regulated by IL37a in the IL-1R8-dependent (overlapping genes) or IL-1R8-independent pathways (the gene in purple area). (**F**) Utilized the InnateDB database to screen for genes associated with the innate immune response. ns, *p* > 0.05 and * *p* < 0.05 vs. the IL-37atg group.

**Figure A5:**
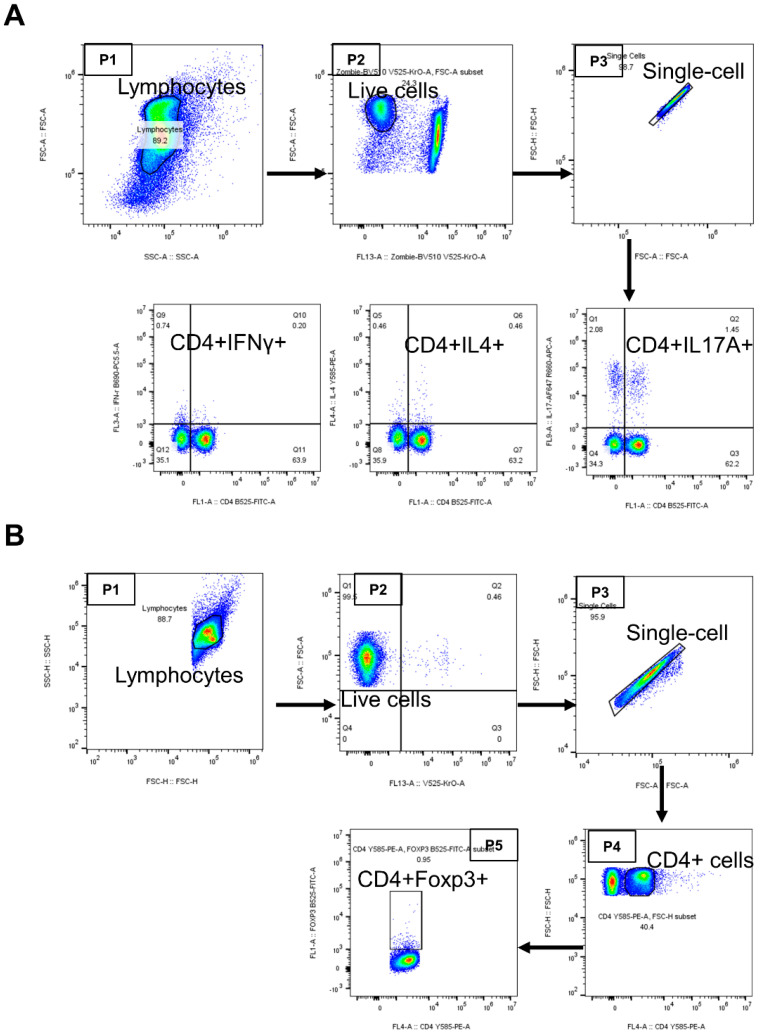
Flow cytometry gating strategy. (**A**) P1 gates the lymphocyte population; P2 gates the live cells; P3 gates the singlet cell population; Q2 gates the CD4+IL17A+ cells; Q6 gates the CD4+IL4+ cells; Q10 gates the CD4+IFNγ+ cells. (**B**) P1 gates the lymphocyte population; P2 gates the live cells; P3 gates the singlet cell population; P4 gates the CD4+cells; P5 gates the CD4+Foxp3+cells.

**Figure A6:**
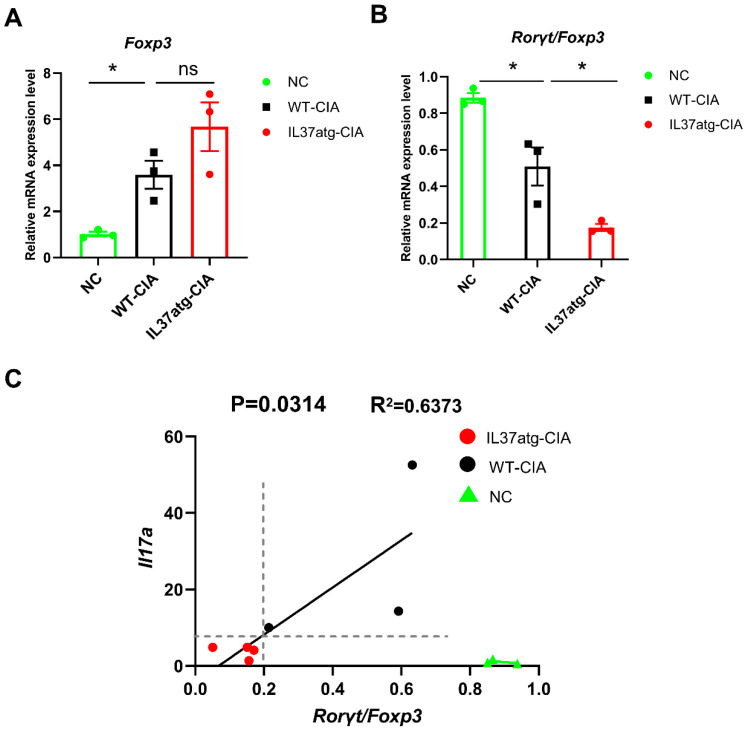
The mRNA level of IL-17a in CIA mice showed a positive correlation with the ratio of Rorγt mRNA to Foxp3 mRNA. (**A**) Foxp3 mRNA expression in single-cell suspensions from draining LNs was measured by qPCR. (**B**) The ratio of ratio of Rorγt mRNA to Foxp3 mRNA was determined. (**C**) Correlation analysis of the relationship between Rorγt/Foxp3 and IL17a mRNA levels. * *p* < 0.05 vs. the WT group.

IL-37 lacks a homologous gene in the mouse genome. For research purposes, transgenic mice that expressed human IL-37 were utilized [8]. IL-37atg and IL1R8-deficient mice were gifts from Professor Damo Xu (Shenzhen University, China) [21]. Male founders were bred with wild-type (WT) female mice by Cyagen Biosciences Inc. (Suzhou, China). The genotypes of mice were determined by PCR when they were 3–4 weeks old. The results of genotypes PCR are shown in Figure A1. Mice from the same litter were age- and sex-matched and utilized in the studies.

### 4.3. Collagen-Induced Arthritis (CIA) Induction and Arthritis Assessment

CIA was induced in specific-pathogen-free (SPF) C57BL/6 mice according to previous reports [45] and the protocol for the CIA model from Chondrex, Inc. (Woodinville, WA, USA). Briefly, mice (8–10 weeks old) were immunized with 100 μg Chicken collagen II (20012, Chondrex, USA) emulsified 1:1 in complete Freund’s adjuvant (CFA) (7032, Chondrex, USA) on day 0 and were delivered a booster injection on day 21 using the identical emulsion. As recommended, an electric homogenizer was used to prepare the emulsion. The progression of arthritis was assessed by visual scoring at three-day intervals following the second immunization. Assessment of arthritis was conducted using the previously outlined methodology [27]. The arthritis grading was conducted by the researchers in a blinded manner. Mice were anesthetized and killed on day 60; the anterior limbs were isolated for micro-CT, and the posterior limb was isolated for histological studies.

### 4.4. Real-Time PCR After Extracting RNA

RNA was extracted using RNAiso Plus (9108, TaKaRa, Kusatsu, Japan). The preparation of complementary DNA (cDNA) involved the use of 1 μg of total RNA and the reverse transcription kit (RR047A, TaKaRa, Japan), following the guidelines provided by the manufacturer. The primer sequences are detailed in Table A1. Real-time PCR reactions were set up and carried out using the Roche 480 System (Roche Diagnostics, Basel, Switzerland). The relative expression levels of the target genes were determined by normalizing their values to GAPDH using the 2^−∆∆ct^ method.

### 4.5. Histological Analysis

After fixation with 4% paraformaldehyde fixation solution and decalcification with EDTA (R20403, Yuanye Technologies, Shanghai, China), the joints of the mice were embedded in paraffin. The thickness of 4 μm tissue sections was stained by hematoxylin and eosin (H&E). Pathologists conducted the evaluation using the established scoring system [46]. The percentage of intra-articular inflammatory cell regions in high-power magnification fields was assessed.

### 4.6. Micro-CT Detection and Image Analysis

The anterior limbs of mice were scanned with micro-CT (viva CT 80, SCANCO MEDICAL, Brüttisellen, Switzerland), and images were acquired at an effective pixel size of 10.4 μm, energy/intensity of 55 kV, 145 μA, and 8 W. Parameters were calculated using CTAn (Burker, Billerica, MA, USA) including the bone volume-to-tissue volume ratio (BV/TV, %), the thickness of the trabecular bones (Tb. Th, pixel), the number of trabecular bones (Tb. N, 1/pixel), and the average distance between trabecular bones (Tb. Sp, pixel) [47,48].

### 4.7. Immunohistochemistry

Paraffin-embedded tissue sections were dewaxed, followed by a 20 min antigen retrieval process at 98 °C using citric acid buffer (pH = 6.0), followed by immunohistochemistry using an assay kit (PV6001, ZSBIO, Beijing, China). The slides were initially exposed to 5% goat serum for 1 h to prevent endogenous peroxidase activity, followed by incubation with primary antibody (A0286 or A16288, ABclonal, Wuhan, China) at 4 °C overnight, followed by washing with PBS for 3 min each. Next, slides were incubated with a goat anti-rabbit IgG polymerizate for 20 min at room temperature. Later, slides were incubated with DAB (ZLI-9017, ZSBIO, China) for 5 to 8 min and redyed using hematoxylin. Images were captured by microscope (Axioplan2 imaging, Carl Zeiss, Oberkoche, Germany).

### 4.8. Flow Cytometry

Single-cell suspensions were prepared from draining lymph nodes (LNs), specifically from inguinal, axillary, and popliteal LNs [49]. To determine cell viability, Zombie dyes (423101, BioLegend, San Diego, CA, USA) were utilized to label the non-viable cells. To determine T-cell subsets in LNs, cells were labeled with PerCPCy5.5 anti-CD4, PE anti-IL-17A, FITC anti-IFN-γ, APC anti-IL4 or FITC anti-CD4, and APC anti-Foxp3. All antibodies exhibit species reactivity to mice. The abovementioned fluorescent antibodies were obtained from BD Biosciences or BioLegend. The flow cytometer (Beckman Coulter, Brea, CA, USA) was set to a fixed flow speed, and a fixed number of events (100,000) were recorded for each sample. FlowJo 10.8 was used to analyze the data.

### 4.9. Th17 Cell Differentiation

Naïve CD4+ T cells from mice lymph node cells were isolated according to the kit (19765, STEMCELL Technologies, Vancouver, Canada), which employs an immunomagnetic negative selection method. Naïve CD4+ T cells were differentiated into Th17 cells with the assistance of the Mouse Th17 Cell Differentiation Kit (R&D Systems, Minneapolis, MN, USA). On day 4 of differentiation, T-cell colonies were observed. The cells were collected and incubated with Cell Stimulation Cocktail (00-4975-93, Thermo Fisher, Waltham, MA, USA) for 16 h. The presence of CD4+IL17A+ cells was then verified via flow cytometry.

### 4.10. Cytokine Assays

Luminex liquid suspension chips from Wayen Biotechnologies (Shanghai, China) were used to detect cytokines in mouse plasma samples, employing the Bio-Plex Pro Mouse Cytokine Grp I Panel 23-plex. Briefly, plasma was placed in 96-well plates containing microbeads and incubated for 30 min. Subsequently, it was incubated with a detecting antibody for another 30 min. Following incubation, streptavidin-PE was introduced into every well, and the plate was left to incubate for an additional 10 min. Finally, the data were acquired and analyzed using the Luminex 200 system (Luminex Corporation, Austin, TX, USA).

### 4.11. Transcriptomics and Differentially Expressed Gene (DEG) Analysis

After inducing collagen-induced arthritis (as previously described in Section 4.3, transcriptome sequencing was carried out on lymph node cell samples obtained from three different mouse genotypes: wild-type (WT), IL-37atg, and IL-37atg/IL1R8-/-, with two mice per group. The sequencing was performed using the Illumina NovaSeq platform (San Diego, IL, USA). Significantly differentially expressed genes (DEGs) with a *p*-value threshold of 0.05 and |log2fold change (FC)| exceeding 1 were identified using the DESeq2 R package (version 1.20.0). To screen for DEGs associated with the innate immune response, we utilized the InnateDB database, which focuses on innate immune genes [11]. Sangerbox was used to generate Euler diagrams to visualize overlapping gene sets [50]. The cluster profile R package was utilized to examine the enrichment of KEGG pathways by differentially expressed genes (DEGs). Specifically, we focused on pathways related to the immune system and signal transduction. The KEGG enrichment results were visualized using a bubble chart generated by Sangerbox [50].

### 4.12. Statistical Analysis

Statistical analysis and data visualizations were conducted with the help of GraphPad Prism Software (version 8.0). The Chi-square test was employed to evaluate the incidence of arthritis, and the arthritis score was analyzed using the nonparametric Mann–Whitney *U* test. Student’s two-tailed t-test or one-way ANOVA was employed for comparisons among two or three groups. A *p*-value less than 0.05 indicated statistical significance.

## 5. Conclusions

**Figure 6:**
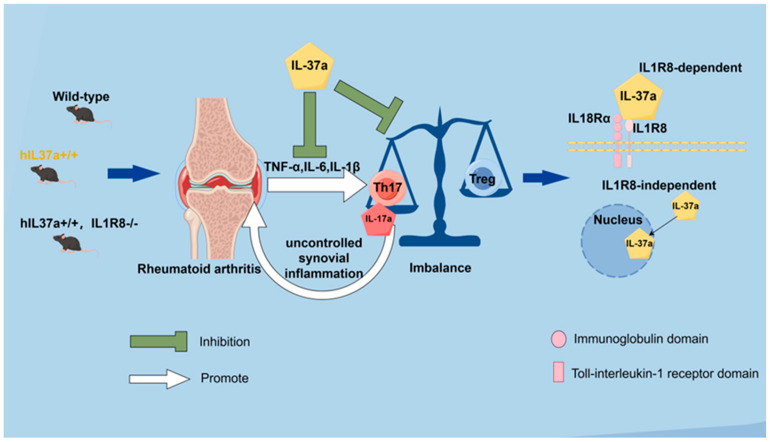
The working model of how IL-37a prevents collagen-induced arthritis. The FigDraw website assisted in creating this visual summary.

In summary, our results demonstrated that human IL-37a can ameliorate the pathological progression of CIA in mice. This protective effect is associated with reduced levels of circulating and joint-localized inflammatory factors, as well as the regulation of T-cell subsets balance (Figure 6). Our research reveals that IL-37a, akin to IL-37b and IL-37d, is a biologically active cytokine capable of modulating inflammatory processes. Notably, IL-37a is a key player in sustaining the delicate Th17/Treg balance, which is of utmost importance to finding new treatment options for rheumatoid arthritis (RA). Furthermore, the IL-1R8 receptor mediates the protective effects of IL-37a against arthritis, highlighting a significant pathway that could be exploited for therapeutic purposes. Further research is required to elucidate the mechanistic role of IL-37a in regulating the phenotypes or functions of other key immunity cells within the context of rheumatoid arthritis (RA).

## Data Availability

The datasets used and/or analyzed during the current study are available from the corresponding author upon reasonable request.

## Appendix A

**Table A1:** The PCR primers listed below were utilized in qRT-PCR.

Gene	Primers	Sequence (5′>3′)
*IL37a* (Human)	Forward	GGGAAACAGAAACCAAAGGA
	Reverse	CCCAGAGTCCAGGACCAGTA
*IL37b* (Human)	Forward	AGCCTCCCCACCATGAATTT
	Reverse	ATTCCCAGAGTCCAGGACCA
*IL37c* (Human)	Forward	AGTGCTGCTTAGAAGACCCG
	Reverse	CCCTTTAGAGACCCCCAGGA
*IL37d* (Human)	Forward	GGGGGAGAACTCAGGAGTGA
	Reverse	ACCTTTGGACCTTCTAAGCAGC
*IL37e* (Human)	Forward	CCCAGTGCTGCTTAGAAGAGA
	Reverse	CCTTTAGAGACCCCCAGGAGA
*Rorc* (Mouse)	Forward	CCGCTGAGAGGGCTTCAC
	Reverse	TGCAGGAGTAGGCCACATTACA
*Foxp3* (Mouse)	Forward	CCCATCCCCAGGAGTCTTG
	Reverse	ACCATGACTAGGGGCACTGTA
*Il-17a* (Mouse)	Forward	TTTAACTCCCTTGGCGCAAAA
	Reverse	CTTTCCCTCCGCATTGACAC
*GAPDH* (Human)	Forward	CTGACTTCAACAGCGACACC
	Reverse	TAGCCAAATTCGTTGTCATAC
*Gapdh* (Mouse)	Forward	GTGTTCCTACCCCCAATGTGT
	Reverse	ATTGTCATACCAGGAAATGAGCTT
